# The Developmental Phases of Zebrafish Myogenesis

**DOI:** 10.3390/jdb7020012

**Published:** 2019-06-02

**Authors:** Samuel R. Keenan, Peter D. Currie

**Affiliations:** Australian Regenerative Medicine Institute, Monash University, Victoria 3800, Australia; samuel.keenan@monash.edu

**Keywords:** muscle, zebrafish, development, primary myogenesis, adaxial cells, growth, secondary myogenesis, external cell layer, evolution

## Abstract

The development and growth of vertebrate axial muscle have been studied for decades at both the descriptive and molecular level. The zebrafish has provided an attractive model system for investigating both muscle patterning and growth due to its simple axial musculature with spatially separated fibre types, which contrasts to complex muscle groups often deployed in amniotes. In recent years, new findings have reshaped previous concepts that define how final teleost muscle form is established and maintained. Here, we summarise recent findings in zebrafish embryonic myogenesis with a focus on fibre type specification, followed by an examination of the molecular mechanisms that control muscle growth with emphasis on the role of the dermomyotome-like external cell layer. We also consider these data sets in a comparative context to gain insight into the evolution of axial myogenic patterning systems within the vertebrate lineage.

## 1. Introduction

In fishes, axial muscle is the most abundant tissue type in the body and functions to produce undulatory locomotion: wave-like contractions along the length of the body that propels the fish forward. Development of the early zebrafish embryo, like that of other vertebrates, involves gastrulation to generate axial and paraxial mesoderm compartments. Axial musculature development (i.e., primary myogenesis) initiates within the paraxial mesoderm, which segments along the anteroposterior axis to form blocks of cells termed somites. Concurrent with segmentation, the paraxial mesoderm undergoes morphogenetic changes to form a series of molecularly distinct cell populations, including the adaxial cells [1,2], primary myotome [3], dermomyotome-like external cell layer [4,5,6,7], endotome [8], and sclerotome [9,10] (Figure 2). Following its initial formation, the myotome undergoes tremendous growth (secondary myogenesis) to reach the adult size (Figure 3).

Zebrafish have provided an excellent model for studying muscle development and growth since the 1960s [11,12]. Early studies mainly focused on the biochemical composition of differing muscle groups and fibre types in fishes, and how these related to their functions. Muscle fibre types are generally described as either slow-twitch, based on possessing slower contraction speeds and being biochemically oxidative for low intensity, high endurance movements; or conversely, fast-twitch due to rapid contraction speeds suitable for high intensity spontaneous movements. Fast twitch fibres fatigue quickly due to being biochemically anaerobic/glycolytic. Additionally, zebrafish slow and fast fibre types are spatially separated to the superficial and deep myotome, respectively [13,14]. This arrangement contrasts to that of amniotes such as chicken and mouse, where fibre types are intermixed in muscle bundles [15,16]. Over the last two decades, molecular studies in zebrafish have provided considerable insight in muscle patterning systems and the signalling pathways that drive differentiation of myogenic precursors towards specific fibre type fates. Intriguingly, such studies show these molecular mechanisms are evolutionarily conserved amongst vertebrates, despite major differences in muscle morphology as the axial muscle of basal swimming vertebrates underwent drastic evolutionary changes for body support [17] and metabolic roles [18], to accommodate the transition from an aquatic to a terrestrial environment.

Studies associated with commercial fish farming in salmon and trout have provided insight into how environmental factors, diet, and hormone treatment, affect muscle growth. These studies demonstrate that teleost species can continue hyperplastic muscle growth (new fibre addition) as adults, resulting in indeterminate growth and final fish size. This observation draws contrast with amniotes, which desist hyperplastic growth during juvenile stages and adults. This raises important questions as to what specific molecular differences exist between vertebrate lineages to regulate muscle growth and secondary myogenesis. Recent advances in muscle stem cell biology have identified the role of the teleost functional equivalent of the dermomyotome, the external cell layer (ECL), in these uniquely teleost secondary myogenic processes. Particular impact was demonstrated in the molecular mechanisms underlying stem cell self-renewal and lineage commitment leading to sustainable tissue expansion. These findings suggest differences between the teleost ECL and the amniote dermomyotome, which we summarise here. While previous studies have generally focused on either primary or secondary myogenesis, we propose a model in which the events occurring during primary myogenesis set the stage for later secondary myogenesis. In this review, we collate recent studies on zebrafish primary and secondary myogenesis, discussing how they fit with existing models, and then integrate molecular and cellular events in primary and secondary myogenesis utilising an evolutionary outlook.

## 2. Primary Myotome Formation in Zebrafish

Prior to segmentation of the paraxial mesoderm, the axial mesoderm differentiates into the notochord, a critical support structure and signalling centre in vertebrate embryos [19]. Paraxial mesodermal cells adjacent to the notochord express myogenic regulatory factors (MRFs), including *myoD* and *myf5* [20,21,22] which induce expression of both slow and fast forms of myosin heavy chain (MyHC) proteins [23]. Expression of MRFs and MyHCs lead to commitment towards a myogenic cell lineage [20,22,24]. Committed cells then form a monolayer against the notochord, and are induced by notochord-derived Hedgehog (HH) signalling, a key factor with many roles in development [25]. HH-induction results in local downstream transcription of *prdm1a*, a repressor of the fast fibre fate, committing the myogenic cell lineage towards a slow fibre fate [1,26]. These slow-specified monolayer cells are termed adaxial cells, which are the first muscle precursors to be specified during post-cranial development, and form the precursors of embryonic slow muscle fibres [24,27,28]. HH signalling is essential for slow fibre specification, as evidenced by their absence or reduction in loss-of-function mutants in HH pathway genes and pharmacological perturbation experiments using the HH-inhibitor cyclopamine [29,30,31,32].

After somite segmentation, the adaxial cells can differentiate into two mutually exclusive slow muscle forms. The more dorsal and ventral positioned adaxial cells, being the vast majority, migrate radially through the somite to a lateral position, forming a monolayer of superficial slow fibres (SSFs) just medial to the ECL [3,14,33]. SSFs are typical slow muscle fibres in that they remain mononucleated throughout development [7]. During early stages of zebrafish primary myogenesis, SSFs are easily distinguished from other muscle fibres due to their horizontal, parallel conformation when viewed laterally. The genetic mechanisms of how adaxial cells migrate and form SSFs are described elsewhere [33,34,35].

The remaining adaxial cells, located at the dorsoventral midline, remain next to the notochord and differentiate in situ forming 2–6 muscle pioneer (MP) cells per somite [1,27,28,36,37,38]. MPs are a form of slow muscle fibres and are the first muscle fibres to differentiate [1,27,28]. MPs are marked by the expression of HH-inducible Engrailed (Eng) transcription factors during differentiation. Eng has notable functions influencing neuronal migration and muscle innervation [37,39,40], highlighting potential functional roles of MPs. Laser ablation experiments of extending MPs resulted in aberrant axonal pathfinding, however axons ultimately reached appropriate targets [41]. Recent findings utilising Eng loss-of-function experiments in zebrafish and chicken complement these initial findings, showing Eng knockdown leads to axon stalling or aberrant pathfinding [40]. MPs have also been suggested to directly give rise to the horizontal myoseptum (HM), a connective tissue sheet structure that dorsoventrally divides the myotome into epaxial and hypaxial compartments [40,42,43]. This hypothesis is largely based on histological observations and analyses of zebrafish mutants for HH and its downstream transcription factor Gli. Such mutants have disrupted development of SSFs and MPs, lack HM development, and have ‘U’ shaped somites instead of normal ‘chevron’ shaped somites [31,44,45]. Interestingly, MPs are known to maintain the somite chevron shape, but do not induce it [46]. Overall, further studies are required to fully elucidate the specific functions of MPs.

In a comparative context, many morphological and histological analyses using genetic markers or TEM cross-sections have identified adaxial cells in teleosts other than zebrafish, including herring [47], trout [21], pearlfish [48], flounder [49], and carp [50]. Additionally, adaxial cells are also known to form in the sturgeon, a non-teleost actinopterygian [51]. Sturgeons lack the development of MP-like cells, with all adaxial cells contributing towards a SSF layer, in addition to relatively delayed HM development when compared to teleosts. To date, adaxial cells have not been characterised in non-actinopterygians and may be a derived trait of this lineage. Somewhat similarly, Eng-positive MPs have only been observed in embryos of zebrafish and medaka [52], both teleost species. The equivalent Eng-expressing muscle cells in tetrapods (mouse [53]; chicken [40]; turtle [54]) and basal gnathostomes (shark [55]) are positioned laterally in the dermomyotome and myotome, and more broadly along the dorsoventral axis relative to the medially positioned MPs of teleosts. Such Eng-expressing cells are phenotypically indistinguishable from surrounding cells, which draws contrast with the distinct flattened phenotype of elongating MPs. These factors indicate fundamental differences between teleosts and non-teleosts in slow fibre generation during primary myogenesis.

### 2.1. Molecular Signalling and Fibre Fate of Adaxial Cells

**Figure 1:**
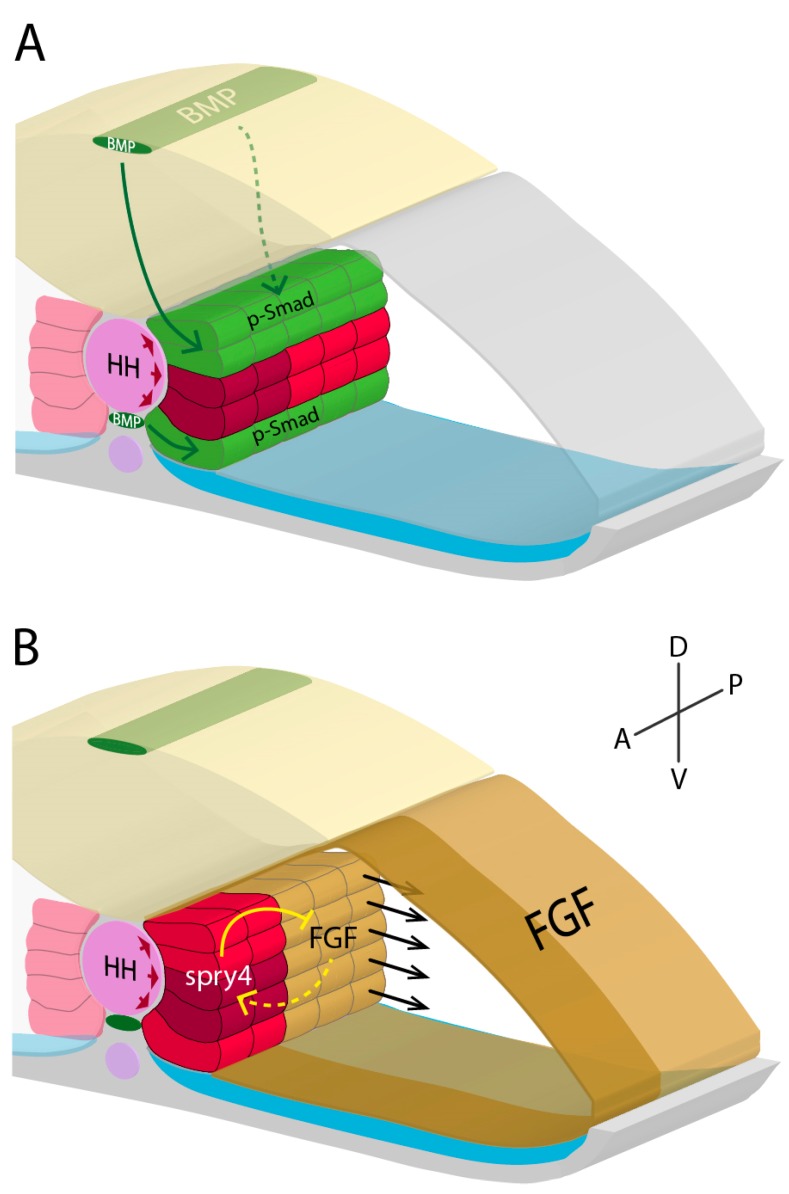
Adaxial cell specification model based on data from Dolez et al. 2011, Maurya et al. 2011, Nguyen-Chi et al. 2012, and Yin et al. 2018. Within the developing zebrafish myotome, coordinated action of HH, BMP, and FGF signalling are necessary for specifying adaxial cells into either SSFs (red) or MPs (dark red). (**A**,**B**) High levels of HH signalling from the notochord (pink) have an inductive effect on adjacent paraxial mesodermal cells, forming medially positioned adaxial cells. (**A**) BMP signalling (green) from the roof plate and hypochord (both dark green) repress MP specification dorsally and ventrally, respectively. (**B**) Additionally, high FGF signalling (gold) in anterior somitic mesoderm induces *spry4* expression in anterior adaxial cells, locally repressing FGF signalling. MP specification in posterior adaxial cells is repressed by FGF signalling. Posterior adaxial cells are the first to undergo lateral migration (black arrows), in response partly to FGF signalling-directed fast fibre differentiation. Together, these distinct pathways generate a 3D signalling system that restricts MP specification to a subset of anteriorly positioned adaxial cells along the dorsoventral midline. Neural keel (yellow); paraxial mesoderm (grey); dorsal aorta (purple); sclerotome (blue).

Adaxial cell specification has been an attractive research topic because of it being a paradigmatic cell fate compartment with unique morphogenesis. In particular, it is intriguing that not all adaxial cells migrate laterally, indicating there are certainly differences in initial genetic specification between SSF and MP precursor adaxial cells. Several distinct signalling pathways have been shown to operate along the different embryonic axes and have been implicated in the formation of MPs and SSFs within the adaxial compartment (Figure 1). As previously mentioned, HH signalling is required for adaxial cell commitment from within the paraxial mesoderm. HH signalling operates along the mediolateral axis, where cells closest to the HH signalling source (notochord) are induced to commit. The post-transcriptionally acting microRNA (miR) miR-214 accentuates the effects of both activator and repressor forms of Gli transcription factors in early segmenting somitic cells [56]. miR-214 increases Gli nuclear trafficking, resulting in accentuated activation of Gli downstream targets in the adaxial cells and accentuated repression in the other somitic cells, thereby sharpening the Gli response and specification of adaxial cells [56]. miR-499 is another fate specifying miR, which is HH-induced and inhibits *sox6*, itself being an inhibitor of slow fibre-specifying genes, thus allowing normal developmental progression towards the slow fibre fate in zebrafish adaxial cells [57].

In addition to HH signalling, bone morphogenetic protein (BMP) signalling also has a role in specifying the adaxial compartment. The dorsal neural tube (roof plate) and hypochord embryonic structures are sources of the BMP ligand *radar* [27,28]. These structures are positioned dorsal and ventral of the notochord, respectively. *Radar*-mediated BMP signalling emanates from these structures to the closest, dorsal-most and ventral-most adaxial cells. Therefore, a dorsoventral signalling gradient of BMP signalling forms within the adaxial compartment, producing low levels of downstream BMP signalling factors (phosphorylated Smad proteins (p-Smad)) in the most midline-associated adaxial cells, and high p-Smad in other adaxial cells [27,28,58] (Figure 1). Knockdown of *radar* function results in an attenuation of the BMP signalling gradient, and subsequently an increase in the number of MPs specified at the expense of SSFs [28], showing BMP signalling (p-Smad) is repressive for MP specification. Maurya and colleagues (2011) identified that p-Smad accumulates within the nuclei of dorsal and ventral adaxial cells and directly represses HH-responsive Eng expression, showing BMP signalling can inhibit downstream HH signalling. Furthermore, such adaxial cells with low HH signalling and high BMP signalling have high levels of repressor forms of Gli, which potentiate the nuclear accumulation of p-Smads and subsequently represses MP fate. Interestingly, the physical structure of cells within the paraxial mesoderm can play a part in adaxial cell specification. The establishment of the BMP signalling gradient is modulated by the extracellular matrix (ECM). LamininC1, an ECM-deposited protein involved in basement membrane formation and cell-to-ECM attachment, helps shape the distribution of BMP signalling [58]. Mutant zebrafish lacking *lamininC1* do not form MPs but are rescued by BMP knockdown. These data indicate LamininC1 reduces the dorsoventral progression of BMP through the ECM from the roof plate and hypochord, and thereby decrease or prevent BMP from reaching the MP precursor space [58].

Finally, FGF signalling has been shown to specify adaxial cells along the anteroposterior axis. Using pharmacological perturbations (FGF-inhibitor SU5402) and FGF signalling loss-of-function experiments, Nguyen-Chi and colleagues (2012) showed that an increased number of MPs were specified at the expense of SSFs within the adaxial compartment. Conversely, zebrafish mutants lacking the FGF signalling inhibitor *sprouty4* (*spry4*) displayed reduced numbers of MPs but normal numbers of wild-type phenotype SSFs [28]. These data highlight that FGF signalling directly inhibits MP specification. Significantly, *spry4* is endogenously expressed in the anterior adaxial cells in response to high levels of FGF signalling, where it then locally inhibits downstream FGF signalling targets including *erm* [28]. Furthermore, expression analyses of *erm* show broad somitic expression initially [35,59], however later is downregulated in the anterior adaxial cells in response to *spry4* induction [28]. This suggests FGF signalling-induced *spry4* generates an anteroposterior gradient of FGF signalling within the adaxial compartment (Figure 1). Following this work, recent findings have suggested that FGF signalling may also act indirectly via its role in fast fibre development [60] to influence adaxial cell specification [35]. Yin and colleagues (2018) generated zebrafish morphants with knocked down function of *ripply1*, a gene required for fast fibre differentiation. Morphants presented a lack of differentiated fast fibres, adaxial cells that did not laterally migrate, and an increased number of MPs. These data suggest FGF-induced fast fibre development is necessary for the lateral migration of adaxial cells, where the SSF precursor adaxial cells are physically forced away from the medial position. This forced migration was thought to prevent HH signalling from further influencing these cells, thereby preventing a MP fate. Additionally, FGF overactivation was shown to both prematurely elongate differentiating fast fibres and increase the velocity of the lateral migration of SSF precursor adaxial cells. Overall, these studies highlight the direct and indirect roles of FGF signalling in specifying cells within the adaxial compartment.

### 2.2. Posterior Paraxial Mesoderm Differentiation

**Figure 2:**
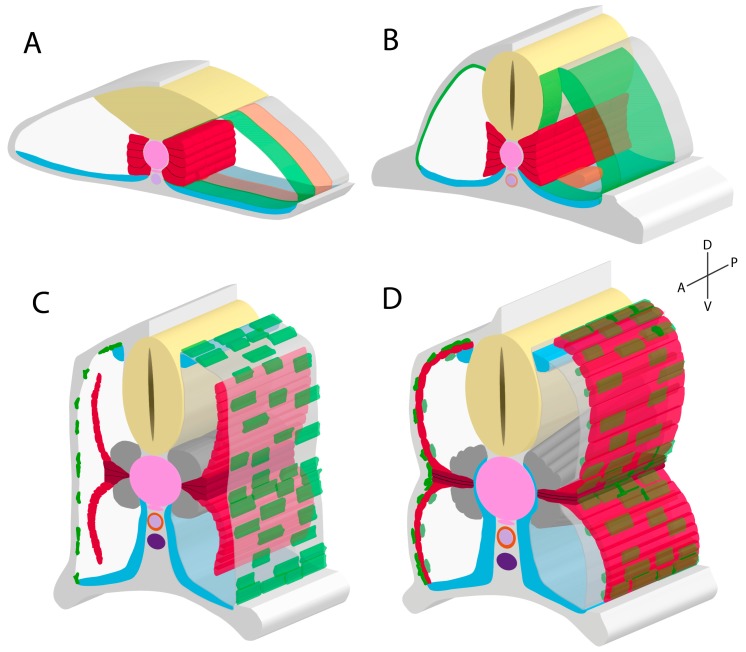
Schematics of 12 hours post fertilisation (**A**, hpf), 16hpf (**B**), 18hpf (**C**), and 24hpf (**D**) zebrafish embryos during somitogenesis and primary myogenesis. (**A**) Paraxial mesoderm (white) is specified to compartmentalise into adaxial cells (red), fast muscle precursors (transparent grey), ABCs (green), endotome (orange), and sclerotome (blue). (**B**) Whole somite rotation progresses, leading to the ABCs migrating laterally and posteriorly, whilst the fast fibre precursors migrate medially and anteriorly. Endotome cells migrate medially to envelop the dorsal aorta (light purple). (**C**) Most adaxial cells migrate laterally through the lateral fast fibre precursors, with some remaining dorsoventral-midline associated and forming MPs (dark red). More dorsal and ventral paraxial mesoderm neighbouring the notochord (pink) is induced to differentiate into MFFs (dark grey). The ECL is established at this stage. In addition to the ventral sclerotome, a dorsal sclerotome region develops [10]. (**D**) By 24hpf, the SSF monolayer has formed, being now lateral to the underlying lateral fast fibres. MFFs, like lateral fast fibres, elongate in an oblique orientation. The ECL participates in secondary myogenesis, generating new fast fibres (light green). Somitogenesis ends with the formation of myosepta, forming a chevron somite shape. Neural keel/tube (yellow); hypochord (pink); posterior cardinal vein (dark purple).

The remaining cells within the somite (i.e., the non-adaxial cells) form three compartments along the anteroposterior axis based on gene expression, generally designated as the anterior, middle, and posterior compartments. Cells of the posterior compartment elongate and form fast fibres of the primary myotome, the middle compartment forms the endotome, and the anterior compartment forms the ECL (Figure 2). The paraxial mesoderm as a whole initially expresses high levels of Raldh2, an enzyme involved in the synthesis of retinoic acid (RA) [61,62]. RA is an inducer of downstream FGF signalling (*fgf8*), which leads to a second wave of MRFs and subsequent production of fast MyHCs and fast fibre fate [60,61]. RA cell receptors are only present within the posterior paraxial mesoderm compartment, meaning fast fibres are only induced here [61]. Interestingly, this second wave of MRFs are induced independently of HH signalling, unlike slow fibre MRFs, reviewed elsewhere [24]. During the early establishment of the posterior compartment, the most posterior and medial cells within this compartment are the earliest to differentially elongate, followed by more distal and anterior cells in a wave-like manner [6,35]. Previously mentioned findings detailed that elongating fast fibres influence adaxial cell specification, however findings exist showing reciprocal effects. HH signalling mutants for *smo* and *prdm1a* demonstrate large delays in fast fibre formation and fast fibre elongation, respectively [35,63], implicating fast fibre elongation occurs in response to adaxial cell lateral migration. Intriguingly, Yin and colleagues (2018) identified that fast fibre precursor cells fuse between the migrating adaxial cells and subsequently increase in size. Mutants for *prdm1a* exhibited a lack of adaxial cell migration and therefore smaller fast fibre sizes. Currently, specific factors involved in this migratory adaxial cell-fast fibre interaction are unknown, however may be related to cadherin proteins due to their roles coordinating adaxial cell migration [33]. These data indicate that slow and fast fibre precursors require one another for normal specification and differentiation during embryonic development. 

Fast muscle fibres have two known variants that are disproportionate in quantity, much like the slow fibres. The lateral fast fibres make up the vast majority and are the stereotypical fast muscle fibres. Specification of these lateral fast muscle fibres requires the aforementioned RA-induced HH-independent wave of MRF expression [60,64]. Physiologically, lateral fast fibres are multinucleated, and make up most of the adult fish body mass. Additionally, these fibres are obliquely oriented when viewing the trunk laterally (Figure 2).

The second form of fast fibres are the medial fast fibres (MFFs), which are distinct fibres located dorsal and ventral to the differentiated and elongated MPs adjacent to the notochord (Figure 2). MFFs later develop to surround the notochord at juvenile and adult zebrafish stages [27,31,58,60]. Distinct functions of the MFFs from lateral fast fibres are unclear. Early specification of MFFs involves indirect-acting physical processes, where the lateral migration of adaxial cells leaves a somitic space located adjacent to the notochord [35]. The most medial cells of the posterior compartment instantly fill this space, and are locally induced by notochord-derived HH to generate HH-dependent MRF expression [24,35]. These MFF precursors are therefore subject to both HH-dependent and HH-independent induction of MRFs. Intriguingly, MFFs also respond to HH signalling in a similar manner to MPs by expressing Eng, but at comparatively lower levels [31,37]. Specific local inducers for MFF fate, however, are currently unknown. MFF fate is known to be notochord-dependent, as MFFs do not develop in the absence of notochord development unlike lateral fast fibres [60], implying that notochord-derived signalling is essential. Knockdown experiments of a notochord membrane factor, collagen XV, lead to more MFFs at the expense of lateral fast fibres [43]. Thus, a decrease in notochord integrity leads to higher notochord-derived signal diffusion. Although HH signalling is not required for initiation of MFF fate [60], it remains possible that differing degrees of HH signalling, such as late-stage or higher expression, may be able to further influence committed fast MFFs. Additionally, MFF fate is known to be FGF-independent, contrary to lateral fast fibre fate. Zebrafish mutants for *fgf8* present adaxial cells that can migrate through the MFF precursors, resulting in normal MFF precursor migration and induction [60]. However, adaxial cell migration cannot continue through the lateral fast fibre precursors, and therefore reduce differentiation of lateral fast fibres. These genetic influencers of MFFs moprhogenesis highlight an unclear specification process. Identifying the particular mechanisms of specification of these cells is key to better understanding the functional role these muscle fibres provide to the teleost embryonic and adult form.

In addition to slow and fast fibres, most teleost species have intermediate forms positioned within a transitional zone between the SSFs and lateral fast fibres of adult stages [65,66]. This fibre type presents itself as a thin layer of pink adult muscle that consists of intermediate levels (relative to fast and slow) of glycolytic and oxidative enzymes ideal for average intensity locomotion, such as feeding or faster cruising speeds [66] (reviewed by Gurevich et al. 2015). How these distinct fibre types develop, such as whether the precursors stay mononucleated or undergo myoblast fusion, is unknown. Perhaps the position of these fibres provides a clue as to how they develop. Intermediate fibre precursors could be influenced by surrounding fast and slow fibres, receiving factors from both to determine their potentially ‘mixed’ fate. Alternatively, teleost intermediate fibres may develop similarly to amniote intermediate fibre type muscles, termed type 2A fibres, which have similar traits to fast muscle fibres that have been influenced by adjacent slow fibres [67,68]. This is supported by observations of teleost intermediate muscle retaining high myofibrillar ATPase activity like that of fast muscle [69].

### 2.3. Primary Myotome Variation in Teleosts

The majority of analyses investigating the teleost primary myotome utilise the zebrafish as the model system. However, there are over 26,000 extant species of teleosts, which display considerable diversity in muscle morphology and fibre type proportions [70]. Previous studies classified teleosts based on their composition of slow fibres at adulthood and the specific ecological niche they occupied [69,71]. In this work, fish were characterised as ‘sprinters’, ‘sneakers’, ‘crawlers’, and ‘stayers’. ‘Sprinters’ such as the stickleback had myotome compositions dominated by fast fibres, a thin layer of intermediate fibres, and an ECL-like structure, but completely lacked slow fibres. Eels are examples of ‘sneakers’, which mainly have fast muscle for explosive attacks but still retain slow muscle. The common dace, which has a generalist lifestyle, is a ‘crawler’. Salmon, in contrast, are a ‘stayer’. In Salmon, slow fibres comprise the vast majority of its myotome, and are suited for their high endurance migrations upstream rivers. In addition to these classes, there are morphological outliers such as tuna, which are considered some of the fastest swimming fish. Tuna myotomes mainly consist of fast fibres, with slow fibres heavily enveloping the HM, and its lateral surface being covered by fast fibres [72]. This internal location of the slow fibres is opposite to that of zebrafish, raising important questions regarding adaxial cell migration in tuna. Finally, recent research of the adult ocean sunfish shows an extreme example of teleost muscle development. Sunfish have no axial musculature or caudal fin [73], despite being the heaviest living teleost. Rather than axial muscle undulation (like in zebrafish), sunfish movement occurs via a modified dorsal and anal fin, with a pseudocaudal fin acting as a rudder. Despite this exceptionally unusual form, young sunfish can attain cruising speeds similar to one of the fastest swimmers, the marlin.

### 2.4. Development of the External Cell Layer

The anterior paraxial mesoderm specifically expresses the transcription factors Pax3, Pax7, and Meox1, genetic markers for the amniote dermomyotome [4,5,60,74,75]. Cells within this anterior compartment form an epithelial monolayer along the anterior margin of the somite. These monolayer cells are termed anterior border cells (ABCs [6], or row one cells [5]). FGF signalling appears to regulate the relative cellular contributions of the anterior and posterior somitic compartments, and thereby specify the number of ABCs that form. Yin and colleagues (2018) showed complete somitic FGF signalling inhibition leads to ectopic ABCs being specified, and conversely FGF overactivation specifies ectopic fast fibres at the expense of ABCs. An additional specification factor, Mesp-b, normally involved in forming somite boundaries during segmentation, is necessary and sufficient for ABC development, as it induces the expression of *meox1* within the anterior somite and inhibits anterior somite myogenesis independent of *meox1* [76]. Live imaging and lineage tracing studies demonstrate that ABCs undergo an extraordinary set of movements where they rotate laterally to occupy the lateral-most surface of the somite [5,6]. During this process, some of the non-adaxial posterior compartment cells also migrate, filling the vacant space left behind by the rotated anterior compartment cells. These events demonstrate the somite as a whole undergoes drastic spatial rearrangements, changing from anteroposterior to mediolateral orientation. The ABCs locate just lateral to the SSF layer and form the ECL (Figure 2). The ECL is a highly proliferative population of self-renewing muscle stem cells that is the teleost functional equivalent to the amniote dermomyotome, contributing new fibres to the primary myotome and driving secondary myogenesis (discussed below). An undifferentiated cell layer external to the myotome (i.e., an ECL-like layer) has been identified in various teleosts including zebrafish [77], sea bass [78], herring [79], sea bream [80,81], and pearlfish [82,83], suggesting its broad conservation among teleosts. HH signalling is required for development of the amniote dermomyotome [84,85], and possibly the teleost ECL [74], however development of both structures differs considerably. The most dorsolateral cells of newly-formed somites differentiate in situ and do not undergo a teleost-equivalent ABC rotation event [85].

Interestingly, the development of the ECL is related to the formation of a separate somitic compartment, the endotome. Using both Kaede (photoconvertible green to red fluorescence) and single cell fate mapping strategies, Nguyen and colleagues (2014) demonstrated that in between the anterior and posterior compartments, a middle compartment of cells emigrate medially and contribute to endothelial structures (Figure 2). Unlike the ABCs, endotome cells do not express *meox1*, and migrate to the dorsal aorta where they are involved in formation of haematopoetic stem cells (HSC). Zebrafish mutants that lack *meox1* intriguingly display an expansion of the endotome at the expense of ABCs, leading to increased HSC production. Given the deficit in ABCs, Meox1-deficient fish also exhibit reduced ECL-derived secondary axial myogenesis as well as deficits in appendicular and hypaxial muscle formation [8]. *Meox1* overexpression experiments demonstrate an ECL marker, Pax7, was induced to express in endotome-derived endothelial cells despite these cells having already committed [8]. These findings demonstrate that the zebrafish anterior and middle somitic compartments include molecularly distinct populations of cells that form the ECL and endotome, respectively, and these fates are regulated by expression of *meox1*. Comparatively, amniote vascular endothelial cells have been implicated as arising from the dermomyotome [86,87], suggesting an endotome-like intra-somitic subcompartment may develop in a similar manner in amniotes as teleosts, however, to date this has not been investigated. Additionally, HSCs are initially generated within the dorsal aorta of all vertebrate embryos studied [88,89,90,91], further supporting the hypothesis that the specification and function of the endotome may be highly conserved.

## 3. Secondary Myogenesis and the Role of the External Cell Layer

The compartmentalisation of the zebrafish somite to form the primary myotome, ECL, endotome, and sclerotome is essentially complete by 24hpf [9]. At this stage, axial muscle can contract to produce movement, and tendon-like myotendinous junctions have formed [10], together mediating movement. Like other vertebrate systems, the zebrafish embryo undergoes secondary myogenesis; a rapid upscaling in size to eventually reach adult form, while retaining the general developmental framework from primary myogenesis. In particular, teleost myotome cross-sectional area (CSA) and length increase dramatically, resulting in axial muscle tissue equalling to 40–60% of the total body mass in the adult [92].

Muscle growth is both a polygenic and multifactorial trait, being influenced by many environmental factors throughout the life of the animal. For example, factors including diet [93,94,95], environmental temperature [96,97], season [98,99], oxygen availability [100,101], and exercise [102,103] have all been shown to influence muscle growth. Additionally, suboptimal factors that compromise primary myogenesis such as relatively low water temperature can reduce later secondary myogenesis potential [65]. Advances in salmonid muscle growth rates have identified that rapid growth can lead to problems with flesh quality due to changes in muscle structure and cellularity, although the biological underpinnings of these changes are unknown [104].

Remarkably, the teleost ECL not only drives juvenile muscle growth, but also persists well into the adult stage of zebrafish development. This enables zebrafish to continue secondary myogenesis into adulthood, resulting in indeterminate growth and final fish size [3,65,105]. Tissue growth requires a balance in activity between precursor stem cell self-renewal and differentiation towards the tissue type [106]. In zebrafish, a population of muscle stem cells that give rise to additional axial muscle fibres has been shown to reside and proliferate in the ECL. The zebrafish system therefore retains an eternal population of these self-renewing muscle stem cells, giving rise to lifelong secondary myogenesis. Recently, the molecular mechanisms driving ECL stem cell dynamics have been described and provide insight into the regulation of stem cell fate [8,106]. In addition to ECL muscle stem cells, there are satellite stem cells dispersed in the myotome that contribute towards muscle regeneration [5,107].

### 3.1. Mechanisms of Muscle Growth

**Figure 3:**
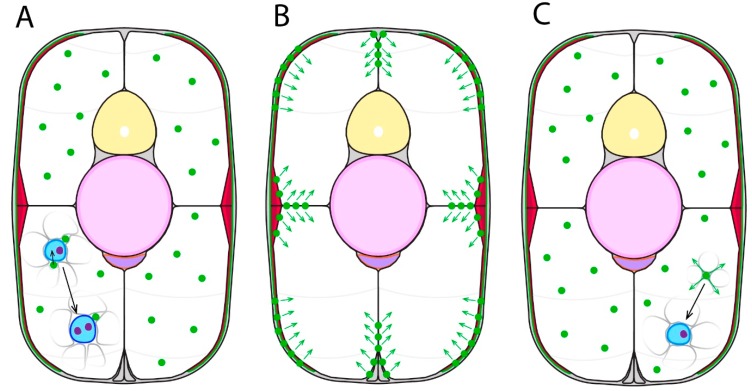
Forms of secondary myogenesis in zebrafish. Schematics represent a transverse section through an early larval stage zebrafish trunk. (**A**) Hypertrophy involves the fusion of myogenic progenitors (green) to existing immature muscle fibres (blue), increasing the number of nuclei (purple) in the muscle fibre. (**B**) Stratified hyperplasia involves new muscle fibres forming at the lateral-most surfaces, either from the ECL, myosepta, or slow fibres. (**C**) Mosaic hyperplasia involves myogenic progenitors throughout the myotome differentiating into new immature muscle fibres.

Within vertebrates, the process of muscle growth can proceed via combinations of two broad strategies [65]. Hypertrophic muscle growth is an increase in the size of established muscle fibres, and hyperplastic muscle growth is the addition of new muscle fibres from muscle stem cell differentiation (Figure 3). Hypertrophic muscle fibres increase in CSA and length by the addition of more myoblast nuclei. Myogenic progenitors situated on the periphery of a muscle fibre directly fuse within the muscle fibre, increasing nuclei number [3]. Recent zebrafish studies have shown muscle fibre CSA directly correlates with age. In adult zebrafish, secondary myogenesis-associated muscle fibres with small diameters are newly formed, and larger diameter muscle fibres are older [106]. Additionally, newly formed muscle fibres that arose in older zebrafish had increased CSA and length compared to younger zebrafish, with characteristically low nuclear composition in new muscle fibres for both age groups. These findings indicate different developmental stages of zebrafish may have distinct hypertrophic mechanisms. Further aspects of teleost hypertrophic growth are reviewed elsewhere [3,108].

Hyperplasia of the myotome initiates with the proliferation of muscle stem cells, which soon after commit to a myogenic pathway and differentiate into new muscle fibres. Within teleosts, hyperplastic growth can be identified by simply counting the number of muscle fibres present within the myotome, which increases with age [106,109]. Two modes of hyperplasia are known to function within the zebrafish, which mainly differ in their spatial origin within the myotome. Stratified hyperplasia generates new muscle fibres in a layer conformation from discrete regions of the myotome that result in a ‘gradient’ of fibres with differing age and CSAs. Zebrafish analyses using muscle-specific fluorophores with differing maturation rates revealed that new muscle fibres were generated mainly in stratified hyperplastic growth zones located within the ECL at the lateral edges of the myotome [106]. Older muscle fibres were located deeper within the myotome. At the end of somitogenesis, teleosts tend to primarily utilise stratified hyperplasia. As the teleost reaches maturity, the efficiency of stratified hyperplastic growth gradually reduces, however still remains functional post-maturity [106]. Mosaic hyperplasia produces new muscle fibres dispersed throughout the musculature, resulting in a mixed arrangement of small and large muscle fibres. Mosaic growth zones are generated by populations of myogenic progenitors located throughout the myotome, with some being suggested to associate with mature muscle fibres [3]. This process occurs in the majority of teleosts studied, at a time after the initial stratified hyperplastic growth of the SSFs [7]. Mosaic hyperplasia is generally reduced in smaller fish species such as zebrafish, rather being of interest for investigating growth in larger commercial fish species [65]. Interestingly, a unique form of hyperplasia exists amongst teleosts, as demonstrated by the toadfish, which is capable of splitting existing muscle fibres to generate new fibres [110]. How the distinct fibre types evident in the adult form relate to the embryonic pattern described above, and how different muscle cell fates are specified during post embryonic growth remains to be resolved.

Like many teleosts, zebrafish are known to utilise both hypertrophy and hyperplasia at hatching stages (72hpf) displaying oriented swimming and foraging behaviour, through to adulthood [3,111]. This conclusion was based on sampling of aging muscle fibres, for counts of new muscle fibres and nuclei, and measurements of CSA and length [3,109]. Fate mapping experiments support this concept, as at 72hpf, Pax7-expressing muscle stem cells begin contributions to muscle growth, as well as some migrating deeper within the myotome [6,112]. Generally, within zebrafish, hyperplastic growth is utilised lifelong but more so during larval stages. Hypertrophic growth then dominates during juvenile and adult stages [3,65,105]. This is similar to amniotes, with the major exception that hyperplastic growth completely ceases postnatally (with a few exceptions [3,113]). Pax7-expressing muscle stem cells within the mouse dermomyotome are necessary for any embryonic or foetal myogenesis, in conditional ablation contexts [114,115]. Consequently, amniotes rely exclusively on hypertrophy for adult muscle growth, having their full complement of muscle fibres by birth [3,116,117].

### 3.2. External Cell Layer Dynamics

The growth of specific fibre types within zebrafish are through distinct processes. The SSF layer initially undergoes stratified hyperplasia at its most dorsal and ventral apices, and the more dorsoventral midline-associated SSFs follow after [7,77]. More knowledge exists for the fast fibre type, with lineage tracing of Pax7-expressing muscle stem cells in zebrafish showing fibres are derived from the ECL [5,6], and likely for pearlfish and trout also [48,118]. Normally, muscle stem cells within the ECL have not committed to the myogenic pathway; however, upon commitment in vitro, they downregulate early myoblast markers while upregulating differentiation markers such as myogenin, forming myotubes in culture [119]. These ECL muscle stem cells migrate in between fibres of the SSF layer, and differentiate at the lateral surface of the lateral fast fibres [7]. Interestingly, the inhibition of myogenic pathway signals such as *myf5* and *myod* leads to an accumulation of undifferentiated muscle stem cells within the ECL [75], suggesting such cells can still self-renew but cannot migrate and differentiate. Studies using trout have shown precursors of lateral fast fibres mainly originate from a posterior lip of the ECL, which migrate medially and then anteriorly leading to differentiation, similar to events of the whole somite rotation [120]. Similarly, it is particularly interesting that higher levels of ECL muscle stem cell migration and differentiation events occur at the dorsal and ventral apices of the myotome relative to other regions in teleosts [7,48,118], which likely reflects ECL phenotype.

Nguyen and colleagues (2017) mapped ECL cell distribution in zebrafish. At 24hpf, ECL cells are evenly distributed. However, using continuous time-lapse imaging methods, they discovered this distribution changes at 72hpf. ECL cells undergo migration, localising along the vertical myosepta (VM) and less so with the HM. Additionally, a distinct cellular pool of Pax3a-positive cells was positioned deeper within the myotome, likely operating for mosaic hyperplasia. ECL-derived cells neighbouring the HM were found to migrate to the nearby VM, however not vice versa. Of interest to growth mechanisms, the migration of cells from the ECL into the myotome only occurred at the VM, and not the HM. All ECL proliferative events were associated with the myosepta, with a large bias towards the VM, revealing additional novel functions of these peculiar structures as self-renewing niches for muscle growth. Proliferative events were identified as either planar, producing daughter cells that remain on the myosepta; or asymmetric, producing one daughter on a myosepta and one within the nearby myotome. The deep myogenic progenitor pool was also shown to undergo VM-associated planar and asymmetric divisions [106]. Since ECL-derived cells align themselves with myosepta which traverse through the myotome, it is plausible that the myosepta serve as guide surfaces for such cells to reach the more medial myotome [82], leading to more distributed secondary myogenesis. Finally, it would be particularly interesting to identify whether these ECL dynamics perform in the same manner in teleost species with dorsal and ventral lips, like trout [4,118], with the hypothesis that there may be even higher levels of dorsal and ventral myotome secondary myogenesis.

Knowing the migration patterns and fates of the ECL-derived cells, it is important to identify the molecular mechanisms underpinning these processes, particularly how such cells self-renew or commit to myogenic pathways. Genetic analyses in recent studies identifying genes related to the ECL focused in on *meox1*, due to its aforementioned inductive expression of the ABCs during early somite patterning [4,5,8,60,74,75]. By 48hpf, *meox1* expression becomes localised to HM-associated ECL-derived muscle stem cells [106]. Intriguingly, Meox1 inhibits *ccnb1*, a gene directing the G2-phase of the cell cycle [106,121,122]. In zebrafish *meox1* mutants, uninhibited *ccnb1* activity was shown to tip the balance of ECL muscle stem cells towards myogenic-committed progenitors at the expense of self-renewing stem cells, leading to ectopic differentiation. Therefore, in addition to its role in somite patterning, *meox1* is required for ECL muscle stem cell self-renewal via a cell cycle arrest effect. In addition, these effects were shown to lead to further downstream positive regulation of *meox1* expression within the ECL-derived muscle stem cell population, stabilising the subsequent self-renewal status. ECL muscle stem cell numbers in *meox1* mutants were reduced to around 40% of that in *meox1*-positive siblings at early embryo stages, however, recover back to normal cell number and density after these stages. Additionally, the number of HM- and VM-positioned ECL muscle stem cells in the *meox1* mutants progressively decreased as the fish aged, with the HM population eventually completely exhausting, unlike in *meox1*-positive siblings [106]. These data therefore detail that Meox1 activity is critical for the uniquely teleost trait of eternal stratified hyperplastic secondary myogenesis within the ECL. It would be interesting to identify how the homologue of *meox1* functions in non-teleost models for secondary myogenesis. Findings described here would indicate non-teleosts either do not express Meox1 in the same manner, or Meox1 or a downstream factor do not function similarly to teleosts, resulting in an eventual exhaustion of their dermomyotome-associated muscle stem cell population. In addition, it is currently not known what specific signals are necessary to induce the ECL self-renewing population to commit to myogenesis.

**Figure 4:**
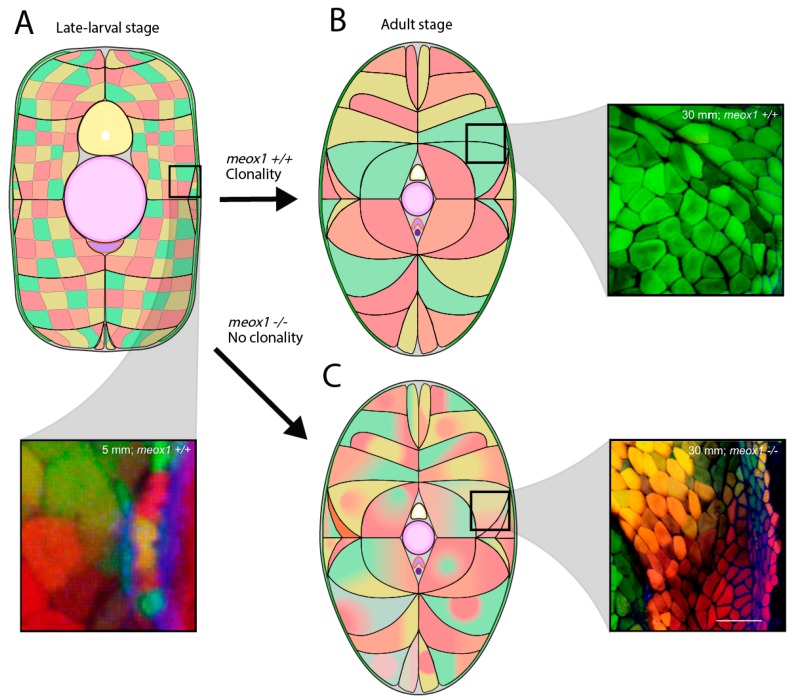
Schematic representing clonal dominance during the early stages of zebrafish secondary myogenesis, using the Musclebow system. (**A**) The late-larval stage myotome displays muscle fibres of different muscle stem cell origin, represented by different colours. Many muscle fibres are within a muscle bundle (black borders denote bundle boundary). (**B**) However, as growth progresses to adult stages, muscle fibres undergo clonal drift, where one muscle stem cell dominates growth within the muscle bundle. (**C**) Mutants for *meox1* display defective ECL dynamics, and muscle fibres fail to drift towards clonality, represented by differing colours within one muscle bundle. Data images reprinted from Cell Stem Cell, 21, Nguyen, P.D. et al., Muscle Stem Cells Undergo Extensive Clonal Drift during Tissue Growth via Meox1-Mediated Induction of G2 Cell-Cycle Arrest, 107-119, 2017, with permission from Elsevier.

A fundamental question regarding secondary myogenesis has been how exactly do ECL-derived muscle stem cells physically contribute towards the complex muscle fibre arrangement observed in adult fish. Recent studies discovered zebrafish secondary myogenesis displays clonal drift [106]. Clonal drift is the transition from an initial diverse population of muscle stem cells all randomly contributing to muscle growth, to relying on a small number of dominant clone populations to generate further muscle growth (reviewed by Sutcu & Ricchetti 2018). This phenomenon has also been observed for other tissue types [123]. Nguyen and colleagues (2017) utilised the Musclebow transgenic system that generates fluorescently-labelled muscle cells with randomised colours, to show that embryonic to mid-larval stage transgenic fish display small, newly generated muscle fibres of unique origin, represented by different colour fibres. Late-larval to adult stage fish displayed the equivalent new fibres, but also large bundles of clonally-drifted differentiated muscle fibres, represented by a single colour (Figure 4). This demonstrated early zebrafish stages display stochastic muscle stem cell activity to contribute to muscle growth, with the clonally-dominant stem cells possessing superior self-renewal, thereby resulting in these to dominate growth of the myotome. The underlying genetic mechanisms for this clonal drift phenomenon were shown to be controlled by *meox1*. Musclebow analyses in combination with zebrafish mutants for *meox1* displayed a continuation of the initial stochastic stem cell profile in adulthood, as evidenced by different coloured neighbouring muscle fibres without ever resulting in clonal drift. This was explained by the HM-located population of self-renewing ECL-derived muscle stem cells diminishing over time in *meox1* mutants, due to a high turnover rate and ectopic differentiation, eventually leading to no population and no clonal dominance [106].

Previous authors have described the ECL as the teleost functional equivalent to the amniote dermomyotome [4,5,6]. Both structures constitute of a muscle stem cell layer at the external surface of the myotome that contributes towards secondary myogenesis [4,7]. Expression data from zebrafish, trout, mouse, and chicken also shows both structures express and require *pax3*, *pax7*, and *meox* for their development [4,5,60,74,75]. Although, it is interesting to note the dermomyotome is the initiator of both primary and secondary myogenesis in amniotes [84,85], unlike the teleost ECL which is only the latter, highlighting an important functional difference between the dermomyotome and ECL. In addition, as previously described, the ECL eternally persists and continues contributing towards secondary myogenesis, unlike the amniote dermomyotome which dissociates [124]. Based on ECL dynamics described above [106], it would be likely this difference in persistence is associated with genetic differences in the *meox1* and *ccnb1* pathway controlling muscle stem cell maintenance and commitment.

## 4. Conclusions

Considerable research has highlighted the early genetic specification of zebrafish somitic compartments, and how this leds to muscle groups with varying fibre type (primary myogenesis). This research has identified that the associated molecular signalling pathways are highly conserved with amniote systems, however distinct cellular structures, such as the adaxial cells, are generated during primary myogenesis. The specification of the adaxial cell compartment is complex, and presents a model system for understanding how various genetic mechanisms can integrate to specify cell lineages. These cell lineages directly influence the primary myotome structure and function. For example, the development and evolution of adaxial cells contributes to the teleost morphology of spatially separate muscle fibre types, most obviously represented by the SSFs. The MPs may have a role in primary myotome innervation, however further study is required to identify the specific functions and contributions of these cells. In addition, MFFs and intermediate fibres have unclear molecular specification, highlighting additional gaps in understanding of zebrafish primary myogenesis. It is also intriguing that during primary myogenesis, molecular crosstalk exists between two different fibre type precursors, in that migrating adaxial cells and lateral fast fibres require one another for specification and differentiation. This is a concept not well studied in muscle development literature, and would be of high interest for further investigation.

A fascinating trait of teleost secondary myogenesis is that the ECL contributes new muscle fibres eternally, unlike the amniote dermomyotome. Newly-identified morphogenetic mechanisms of ECL cells contribute to our understanding of this teleost-specific phenomenon. Muscle stem cells are initially generated uniformly within the ECL during early secondary myogenesis, and may later migrate towards and along myosepta into the myotome. Populations are formed adjacent to the myosepta, particularly the VM, where they locally self-renew and differentiate into new muscle fibres. Stratified hyperplastic muscle growth from these populations undergoes clonal drift, with the most proliferative muscle stem cells dominating hyperplastic contribution. Muscle stem cell populations can maintain their self-renewing state or proceed to undergo muscle fibre differentiation, via *meox1*-mediated regulation of the cell cycle. Alteration of this cell cycle genetic pathway resulted in an eventual exhaustion of the ECL-derived muscle stem cell populations, altering clonality and eternal hyperplastic growth dynamics. Intriguingly, *meox1* is also necessary for initial developmental specification of the ECL during somitogenesis. These are interesting findings as they detail connections and dependencies between somitogenesis, primary myogenesis, and later secondary myogenesis. This therefore represents an example of direct developmental integration between embryonic and adult teleost life stages, which is a concept not particularly well portrayed in myogenesis literature. Moreover, in contrast to teleosts, a lack of coordinated genetic mechanisms to maintain a self-renewing muscle stem cell population likely explains why hyperplastic growth ceases postnatally in the amniote dermomyotome. Future study of the genetics underlying muscle stem cell populations, particularly the unknown but likely important role of extrinsic factors, would improve our understanding of secondary myogenesis by linking together environment, genetic regulators, cell dynamics, and overall muscle phenotype. Finally, although considerable understanding exists for patterning of the zebrafish primary myotome, there is scope for further exciting molecular discoveries for secondary myogenesis, with recently discovered ECL dynamics representing only the beginning.
